# The Abundance and Dynamics of Small Mammals and Their Predators: An Editorial

**DOI:** 10.3390/life14010041

**Published:** 2023-12-26

**Authors:** Ignasi Torre, Linas Balčiauskas

**Affiliations:** 1 Small Mammal Research Area and BiBio Research Group, Natural Sciences Museum of Granollers, Francesc Macià 51, 08402 Granollers, Spain; 2 Laboratory of Mammalian Ecology, Nature Research Centre, Akademijos 2, 08412 Vilnius, Lithuania; linas.balciauskas@gamtc.lt

Small mammals (rodents and insectivores) represent an eclectic group of numerous species of different phylogenetic origins that share similar biological/ecological characteristics derived from their small size [1]. This group is considered to play a key functional role in ecosystems worldwide, providing several benefits, such as vegetation and soil regeneration (as seed dispersers and burrowers) and their position at the bottom of food webs, thus playing a central role as both the predators of invertebrates and the prey of medium-sized carnivores and raptors [2]. On the other hand, they are frequently regarded as pests, especially in anthropogenic ecosystems [3].

Indeed, small mammals show complex and interactive top–down and bottom–up regulation of a number of other relevant components of their communities [4]. Being short-lived, in addition to having fast generation times and demographic responses to environmental factors, small mammals are ideal subjects to study population dynamics in time and space as compared to long-lived mammal species. In light of environmental change because of human activities, landscapes and the climate are suffering from alterations, producing range shifts and the restructuration of small-mammal assemblages [5], which are now dominated by opportunistic species [6] with poor conservation value and limited functions, leading to the requirement for new management strategies for the conservation of ecosystems.

The aim of this editorial is to introduce the Special Issue “Abundance and Dynamics of Small Mammals and Their Predators”, which included 10 papers in total (two reviews and eight research papers). Almost all studies were conducted in European countries, with four from Spain [7,8,9,10], two from Lithuania [11,12], one from Greece [13], and one from Latvia [14]. The remaining two studies were conducted in New Zealand [15] and Chile [16]. Regarding the topics covered in this Special Issue, the last two mentioned articles addressed the effects of alien species in ecosystems, three focused on the presence and natural control of potential pest species in agroecosystems, two concerned the historical trends of small mammal populations and the effects on their predators, and one discussed the activity of small mammals along man-made gradients of vegetation affected by predation risk. Two articles focused on individual species: one examined the effects of landscape change on the demography of a shrew, while the other analyzed the reproductive patterns of dormice.

The review by Carolyn King [15] takes a historical approach to the arrival and spread of several alien species in New Zealand. The two islands were accidentally colonized by three species of rats and by the house mouse, and these alien species quickly dispersed because of a lack of competitors and predators. Rather surprisingly, New Zealand did not have any terrestrial non-volant mammals in its endemic fauna; thus, the invasive species took over the vacant niches and assumed new ecological roles, with them experiencing incredible population outbreaks. Unfortunately, they were stronger competitors/predators for the endemic fauna composed of several species of birds, lizards, frogs, and invertebrates, whose populations vanished until extinction in some cases, therefore contributing to the historical decline of New Zealand’s biodiversity. In an attempt to control “uncontrolled” and widespread small mammal populations, humans introduced several predators (e.g., stoats and weasels), which also quickly spread across the territory, preying not only on alien species but also—and sometimes mostly—endemic species, thus causing additional damage to the endemic communities. This is a terribly sad example of the accidental effects caused by humans during the colonization of new lands and how the effects can last for centuries despite efforts to actively fight against them. The New Zealand government started an eradication plan to eliminate all predators from the country by 2050, hardly a manageable plan!

In their review, Balčiauskas and Balčiauskienė [12] studied changes in small mammal communities over five decades (1975–2021) in one of the Baltic countries, Lithuania. Gathering an enormous dataset of nearly 60,000 small mammals, which were trapped or found as owl prey, they constructed the composition of communities and their variation over time. Two forest-dwelling rodents were dominant throughout the study, the bank vole and the yellow-necked mouse, with them showing similar proportions in either trapping or owl pellets. The authors showed that the small mammal diversity increased after the 1990s, which was interpreted as a response to fast landscape changes that occurred after the country reached independence from the Soviet regime.

The European rabbit is an invasive species in South America, but it has been coexisting and interacting with native species in Chile for around 150 years. Highly integrated into food webs, the rabbit built complex interactions as a plant consumer, as a competitor of other endemic herbivores (rodents), and as a prey of predators. Gübelin et al. [16] studied the situation of the rabbits in the food network in a semi-arid area of Central Chile. The study area was set in Las Chinchillas National Reserve, one of the central background areas in South America for the study of the population dynamics of small mammals and their interaction with predators, climate, and vegetation. This study used a long data series spanning 36 years (1987–2022) and considered an extensive food web consisting of 77 species to construct their interactions. Unexpectedly, the rabbit was the species showing a higher number of connections with the network, with it outcompeting the native species. Therefore, the reduction in or eradication of populations of invasive species, such as the aforementioned rabbits, can produce unexpected consequences for the community structure and ecosystem processes if species interactions are not completely uncovered and understood.

Three papers included in this Special Issue are related to the interaction between small mammals and their predators. Avotins et al. [14] studied the population dynamics of small mammals after a monitoring program lasting 25 years (1991–2016) in Latvia and compared the changes experienced by small mammals with those undergone by their own predators. To link owl demography to small mammals, they studied the diet of six owls during the breeding season and monitored their breeding success. The small mammal populations (Microtus and bank voles) showed a significant decline during the study period, and the diet of the owls increased the food niche breath as far as small mammals being less available with time. Regarding the owl populations, the smaller species showed steep declines (*Aegolius funereus* and *Glaucidium paserinus*), but the larger species showed population stability (*Strix aluco*, *Strix uralenis,* and *Asio otus*). The two former species showed more specialized diets based on voles, and the other showed higher diet plasticity, with them not being affected by vole population dampening.

Studying the diet of some generalist predators is a useful indirect technique to gather information on the spatial and temporal abundance of their prey. Bontzorlos et al. [13] used the diet of the barn owl, an open habitat predator, to interpret the spatial changes in relative abundance experienced by some species of voles—considered agricultural pests—in the Thessaly plains (central Greece). During three breeding periods (2003–2005), they took 10,000 pellets from 31 barn owl territories, describing land uses and other relevant variables within 2 km buffers. They identified nearly 30,000 prey, almost all small mammals (98%) of 15 different species, and showed that soil texture and composition were more relevant than land uses for explaining the distribution of four vole species. The authors claim to consider these—almost ignored—variables in spatial analyses in intensive agricultural land.

Small mammals can be seen as agricultural pests, and in several countries, they have been persecuted and exterminated by using poison to prevent crop damage. However, this practice has several negative impacts on the environment, and it is forbidden under European Community policies. Therefore, alternative practices for controlling small mammal pests need to be implemented. The presence of birds of prey and owls can be an effective method of biological rodent control. Jareño et al. [10] found that nest boxes occupied by common kestrels (*Falco tinnunculus*) and barn owls (*Tyto alba*) had a negative influence on common vole (*Microtus arvalis*) densities within distances of up to 180 m. Vole densities were gradually re-established beyond this distance. The most significant reduction in vole populations occurred at the end of the breeding period, and the presence of a greater number of fledglings decreased the probability of vole presence over longer distances. Therefore, increasing the number of nest boxes for mouse-eating birds could be a feasible measure for the biological control of the common vole in Mediterranean ecosystems. However, the economic profitability for farmers to install nest boxes without subsidies still requires verification.

Agricultural land is inhabited by several small mammal species which adapt their biological cycles to the anthropogenic environment. Balčiauskas et al. [11] studied the composition of small mammal communities living in 18 fruit and berry farms in Lithuania from 2018 to 2022. In their study, the authors describe several parameters in detail, such as litter sizes and breeding conditions and the population structure (i.e., sex and age). During those five years, they trapped 1936 individuals of 13 small mammal species. As a general pattern, the control non-agricultural sites (i.e., meadows and forests) yielded higher small mammal abundance and richness. Populations of the four common species showed interannual, seasonal, and habitat variations in numbers, but none showed outbreaks nor cyclic oscillations. These results are interesting, bearing in mind the conflicts between biodiversity conservation and agriculture, and can be used in agroecology and sustainable farming.

In the Mediterranean basin, landscape and land-use changes have been identified as significant factors contributing to biodiversity loss. Torre and Díaz demonstrated that the current socio-economic conditions are characterized by the expansion of natural habitats into cultural landscapes [7]. In their study, the authors analyzed the effects of landscape change on the demography and spatial distribution of the greater white-toothed shrew (*Crocidura russula*) within six protected areas from 2008 to 2020. The primary natural habitats observed were scrubland, pinewood, and holm oak woodland. The findings revealed that the rapid encroachment of scrubland and afforestation negatively impacted habitat suitability for shrews, leading to reduced territory occupancy by this species. Consequently, these results suggest that the spatial distribution of shrews is changing at a faster pace due to the rapid landscape changes, surpassing previous expectations based solely on climate change.

Habitat changes have a greater number of effects on small mammal ecology, not only population sizes and spatial distributions. Through shading, vegetation mitigates the effects of changing moonlight levels, and this is important for the foraging of small mammals, as moonlight changes predation and anti-predator behavior. Pasquet et al. [8] investigated the impact of moonlight levels on the activity patterns of three common small mammal species in Mediterranean habitats, examining whether the effects of moonlight were influenced by the presence of tall vegetation resulting from spontaneous afforestation after land abandonment. They found that responses to moonlight were species-specific. Nocturnal wood mouse (*Apodemus sylvaticus*) activity decreased under moonlight conditions independent of vegetation cover. The activity of the greater white-toothed shrew (*Crocidura russula*) remained on the same level independent of moonlight levels. The activity of Algerian mice (*Mus spretus*) increased, as was favored by vegetation cover. These findings indicate that changes in vegetation gradients, influenced by human activities, can create varying fear landscapes, which in turn, may lead to community-level changes in small mammal populations.

Based on an 18-year-long data series, Míguez et al. [9] analyzed the reproductive patterns of the edible dormouse (*Glis glis*) populations in the northeastern region of the Iberian Peninsula. Their findings indicate that maternal body weight influences the average weight of the pups but does not appear to impact litter size. Therefore, there was no trade-off between offspring number and size at birth. The authors did not identify any discernible geographic patterns in litter size based on latitude, longitude, and elevation along a gradient spanning from the Iberian Peninsula to the Pyrenees, therefore discarding hypotheses that (i) larger litters compensate for shorter seasons associated with higher altitudes or northern latitudes and (ii) changes in litter size are influenced by climatic variations (e.g., temperature or precipitation) along latitudinal and/or altitudinal gradients. This investigation holds particular significance within the context of climate change.
